# Sanao decoction for asthma: Protocol of a systematic review

**DOI:** 10.1097/MD.0000000000015313

**Published:** 2019-05-03

**Authors:** Guoming Chen, Yijun Chen, Ziyin Chen, Shuai Gao, Peng Zhang, Honghui Zhang, Yanfen Huang, Yingtong Lin, Li Wei

**Affiliations:** a Guangzhou University of Chinese Medicine; b First Affiliated Hospital of Guangzhou Medical University; c National Clinical Research Center for Respiratory Disease; d Department of Pharmacy, First Affiliated Hospital of Guangzhou Medical University, Guangzhou, China.

**Keywords:** asthma, protocol, sanao decoction, systematic review

## Abstract

**Background:**

Asthma is a chronic inflammatory disease characterized by recurrent attacks of breathlessness and wheezing, which often worsen at night or in the early morning and vary from person to person in severity and frequency. Sanao decoction (SAD), as a traditional Chinese medicine compound, has a long history of clinical application in the treatment of respiratory diseases. Whereas neither systematic nor meta-analysis of randomized controlled articles explain the efficacy of SAD in treating asthma. Therefore, we provide a protocol to evaluate the efficacy and safety of SAD for asthma.

**Methods:**

From the beginning to December 2018, the following electronic databases will be searched for studies in English or Chinese: the Cochrane Library, Embase, PubMed, Web of Science, the Chinese National Knowledge Infrastructure, the Chinese Biomedical Literature Database, the Chinese Scientific Journal Database, and the Wanfang Database. Total effective rate, peak expiratory flow (PEF), forced expiratory volume in 1 second (FEV1), forced vital capacity (FVC), and FEV1/FVC will be measured as primary outcomes. Meta-analysis will be performed using the Stata 15.

**Results:**

This study will provide the current evidence of asthma treated with SAD from the several points including PEF, FEV1, FVC, and FEV1/FVC.

**Conclusion:**

The consequence of this summary will furnish proof to evaluate if SAD is effective in the treatment of asthma.

**PROSPERO registration number:**

PROSPERO CRD42018117923.

## 1 Introduction

Asthma is a chronic respiratory disease usually along with clinical manifestations such as wheeze, shortness of breath, chest tightness, cough, and other variable airflow limitation symptoms.^[1,2]^ According to the latest estimate released by WHO in December 2016, about 235 million people worldwide currently suffer from asthma.^[3]^ Treatment was achieved by inhibiting airway perfusion with inhaled corticosteroids and alleviating bronchoconstriction with bronchodilators, such as inhaled corticosteroid, long-acting β_2_-agonist, short-acting β_2_-agonist, leukotriene receptor antagonist, theophylline drugs, and immunosuppressive agents.^[4–7]^ The standard treatment can only control most symptoms but cannot deal with all asthmatics. However, conventional treatment has been proved to represent either limited efficacy or frequent side effects, for example, mood changes, transient effects, immunosuppression, and side-effect of steroids on the growth of children.^[8–10]^ While new surgical techniques and drugs have improved health, traditional therapies have often failed to treat chronic diseases gratifyingly.^[11]^ Traditional Chinese medicine (TCM) has been used to treat asthma in Asia for centuries and gradually accepted by the world because of its good curative effect and fewer adverse reactions.

Sanao decoction (SAD) is a traditional prescription developed from *Taiping Huimin Heji Jufang* and clinically applied to treat asthma in Chinese medicine. It promotes the dispersing function of the lung, dispelling coldness, eliminating the phlegm, clearing heat, and relieving a cough, so it is usually used to slow and cure the symptom of wheezing. SAD consists of 3 traditional Chinese medicines, namely Ephedrae Herba (ma huang), Armeniacae Semen Amarum (ku xing ren), and Glycyrrhizae Radix Et Rhizoma (gan cao). The main active ingredient of Ephedrae Herba (ma huang) including ephedrine, pseudoephedrine, and volatile oil can dispel coldness to alleviate asthma, and suppress coughing with the assist of the Armeniacae Semen Amarum.^[12]^ Glycyrrhizae Radix Et Rhizoma is added to harmonize the nature of medicines. What's more, Glycyrrhizin contained in Glycyrrhizae Radix Et Rhizoma has antiinflammatory and antiallergic liveness.^[13,14]^ As could be observed from pharmacologic studies, SAD has significant bronchiectatic effect, reduces airway inflammation, and remodels airway.^[15,16]^ Meanwhile, recent evidence suggests that SAD can treat the airway hyperresponsiveness and had remarkable immunomodulatory effects.^[17–19]^ Recently, with the publication of a number of trials on SAD for asthma, they have proved that SAD has good clinical effect.^[20–23]^ There is an urgent need for a systematic review to support the effectiveness and safety of SAD in treating asthma. Hereby, the purpose of the study is to systematically review current available articles to assess the efficacy and safety of the SAD treatment in patients of asthma.

## 2 Method

The systematic review will be developed in compliance with the Preferred Reporting Items for Systematic Reviews and Meta-Analyses (PRISMA) approach and reported adhering to the PRISMA guidelines. It has been registered on PROSPERO (ID: CRD42018117923)

### 2.1 Eligibility criteria

#### 2.1.1 Study characteristics

Only randomized controlled trials (RCTs) will be included instead of case reports, narrative reviews, systematic reviews, or cross-over trials. Studies lacking complete and accurate data will be excluded. Nothing but articles published in Chinese or English will be considered. We will not impose any restriction on publication status.

#### 2.1.2 Participants

According to the diagnostic criteria by National Institute for Health and Clinical Excellence,^[24]^ participants meeting the diagnostic standard of asthma will be involved irrespective of their age, sex, or ethnicity. Patients with other complicating diseases will not be included. Even the patients’ course of disease and severity of illness will be approximately equivalent.

#### 2.1.3 Intervention

In treatment group, SAD will be the sole treatment for patients, while routine western medicines will be used alone in control group. SAD consists of Ephedrae Herba (ma huang), Armeniacae Semen Amarum (ku xing ren), and Glycyrrhizae Radix Rhizoma (gan cao). Modified SAD will also be included as long as it contains the 3 herbs and increases not exceeding 10 herbs. All the formulas involved should keep to the principles of Monarch, minister, assistant, and guide in TCM prescription. There is no restriction on dosage form or mode of administration.

#### 2.1.4 Outcomes

The following primary outcomes will be measured: total effective rate, peak expiratory flow, forced expiratory volume in 1 second (FEV1), forced vital capacity (FVC), and FEV1/FVC. We will consider the 1-year recurrent rate and incidence of side effect as secondary outcomes.

### 2.2 Search strategy

An exhaustive search will be conducted by using the following electronic databases from the beginning to December 2018: the Cochrane Library, Embase, PubMed, Web of Science, the Chinese National Knowledge Infrastructure, the Chinese Biomedical Literature Database, the Chinese Scientific Journal Database, and the Wanfang Database. The keywords include “sanao decoction” and “asthma.” The search strategy for PubMed is summarized in Table 1. Relevant data will also be searched through other sources: hand searching, conference proceeding, International Clinical Trials Registry Platform, and Chinese Clinical Trial Registry.

**Table 1:**
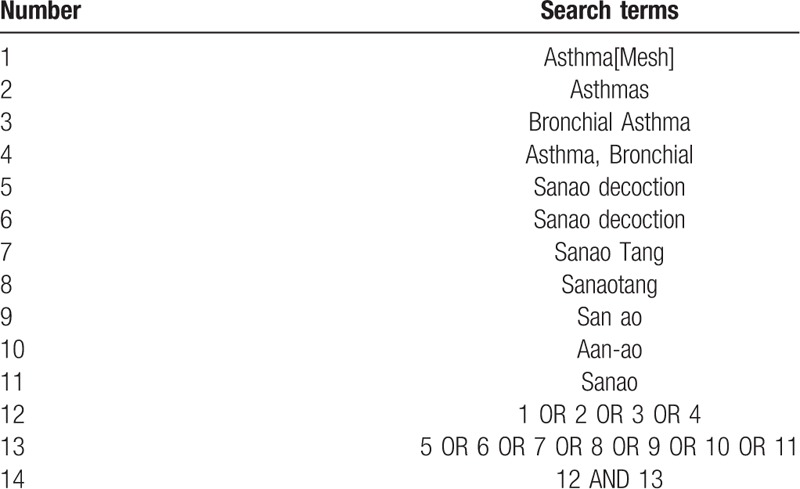
Search strategy for the PubMed database.

### 2.3 Study selection

**Figure 1:**
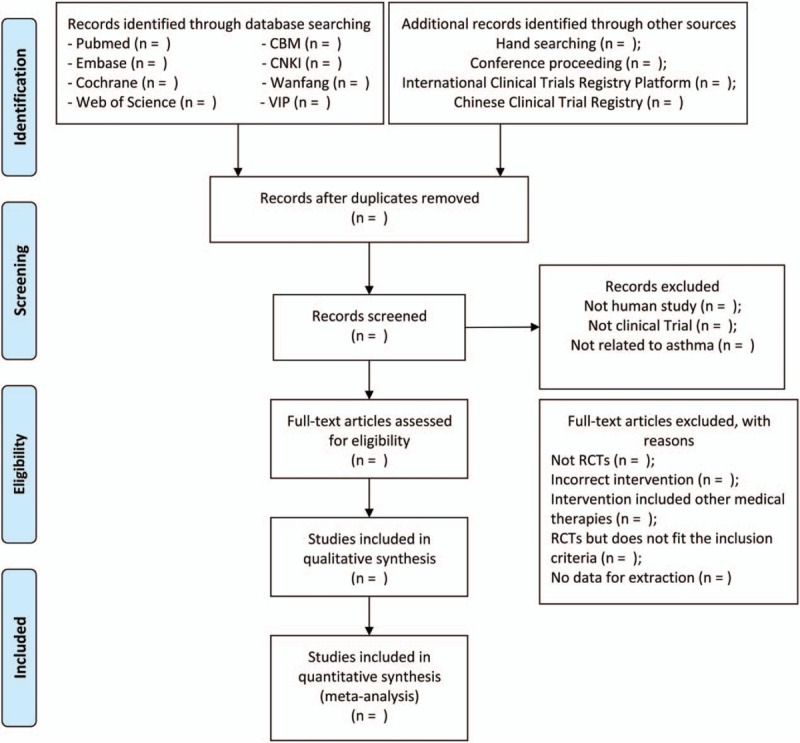
Preferred Reporting Items for Systematic Reviews and Meta-Analyses flow chart of study selection process.

The search results from every database will be combined and the duplicates will be removed by the EndNote X9. According to the inclusion criteria, 2 investigators (PZ and HZ) will select potentially eligible studies by assessing the titles and abstracts independently. Subsequently, 2 investigators will read over the full texts of the included studies and communicate with each other to make a final selection. Some studies will be removed because of below reasons: not RCTs, nonconforming intervention, RCTs but not meeting the inclusion criteria, and no data for extraction. Any divergence will be dealt with by the discussion with a 3rd reviewer (GC). The whole selection process will be presented in a PRISMA flow diagram (Fig. 1).

### 2.4 Data extraction

Two investigators (YH and YL) will perform the data extraction independently using a predefined form, which includes 4 parts: basic information, characteristics of trial subjects, intervention measures, and results of the studies. Again, the 3rd reviewer (GC) will make a final decision in case of discrepancy. If some information is insufficient, we will make an attempt to contact the authors of the original trial. Supposing that the author fails to respond, the study will be discarded and only the available data will be analyzed. The influence caused by the missing data on the meta-analysis results will be taken into consideration.

### 2.5 Quality evaluation on methodology

Taking the criterion in the Cochrane Handbook for Systematic Review of Interventions V.5.2.0 (renovated June 2017),^[25]^ risk of bias will be classified into three categories (low, unclear, and high) independently by 2 verifiers. Overall, the quality assessment will be based on the 7 domains: random sequence, blinding of the participants and personnel, allocation concealment, blinding of outcomes, selective reporting, completeness of outcome data, and other bias. In case of discrepancy, consensus will be reached by a collective discussion.

### 2.6 Statistic analysis

Meta-analysis will be carried through using the Stata 15. The continuous outcome data will be expressed as mean differences, while the dichotomous outcomes will be analyzed by using risk ratios with 95% confidence interval using fixed or random-effect models.

### 2.7 Assessment of heterogeneity

Statistical heterogeneity among the studies will be assessed by *I*^2^ and Chi-squared statistics. Heterogeneity will be considered to be considerable if *I*^2^ ranges from 50% to 100%, for which we will analyze data using a random-effect model. If the tests for heterogeneity have no significant meaning (*I*^2^ ≤ 50%), the fixed effect model will be used.

### 2.8 Assessment of reporting bias

Funnel plots will be conducted to evaluate reporting bias. If potential reporting bias is detected, Begg and Egger test will serve to evaluate the symmetry of the funnel plot and perceive publication bias.

### 2.9 Sensitivity analysis

Sensitivity analysis will be conducted to identify the robustness of the result and detect whether there are any exceptional studies bringing about an evident heterogeneity. Then the particular study will be scrutinized to find the reasons.

### 2.10 Subgroup analysis

Subgroup analyses will be performed to explore the source of heterogeneity according to the following items: duration or severity of asthma: acute, subacute, or chronic, ages of the patients: children or adults, the original or relative prescription of SAD, and duration or dosage of herbal medicine treatment.

### 2.11 Quality of evidence

The dependability of proof will be appraised by the Grading of Recommendations Assessment, Development, and Evaluation (GRADE). The following factors will be taken into consideration: limitations in the design, unaccounted heterogeneity, discrepancy, indirectness of evidence, hidden error, and selective publication. Evidence quality will be rated as high, moderate, low, or very low.

### 2.12 Ethics and dissemination

It is our aim that this review is going to be shed in peer-reviewed journals. Private information from individuals will not be involved in the review, so there is no need for informed consent form. Ethical approval is also unnecessary because this study is not a clinical trial.

### 2.13 Patient and public involvement

Neither patients nor public got involved.

## 3 Discussion

Asthma is a chronic inflammatory airway disease with well-accepted heterogeneity and complex pathophysiologic processes.^[26]^ The pathologic mechanisms of asthma include allergic reaction, bronchial chronic inflammation, airway hyperresponsiveness, abnormality of airway neuromodulation, genetic factor, respiratory viral infection, neural signal transduction mechanism, and airway remodeling.^[23,27]^

The SAD, a traditional Chinese medicine formula, is diffusely used for patients with asthma. It has more advantages than single receptor chemicals in treating asthma with multicomponent and multitarget therapy. These analogous formulas all have commonness in ventilating Fei and superiorities of evidence-based derivation in compliance with multilevel effect evaluation, whose effect pathway was involved in cell structure protection, antiinflammation, antioxidant, and immunoregulation.^[28]^ However, a systematic review of SAD in treating asthma has not yet been published. This systematic review will be the 1st to provide a summary of the current state of proof concerning the effectiveness and safety of SAD in treating asthma. This evaluation will be useful for practitioners and patients with asthma.

## Author contributions

LW is the warrantor of this article. GC and YC drafted the manuscript, and the search strategy was constructed by ZC, SG. PZ, and HZ will select potentially eligible studies and extract data independently. HZ, YH, and YL will evaluate the risk of bias and perform data synthesis. GC will make a final decision in case of discrepancy to make sure that there are no errors during the review. All review authors reviewed, revised, and confirmed the subsequent and final version of the protocol minutely.

**Conceptualization:** Guoming Chen, Yijun Chen.

**Data curation:** Peng Zhang, Honghui Zhang, Yanfen Huang.

**Methodology:** Ziyin Chen, Shuai Gao, Yanfen Huang, Yingtong Lin.

**Project administration:** Guoming Chen, Li Wei.

**Resources:** Guoming Chen, Yijun Chen, Li Wei.

**Supervision:** Guoming Chen.

**Writing – original draft:** Guoming Chen, Yijun Chen, Ziyin Chen, Shuai Gao, Peng Zhang, Honghui Zhang, Yanfen Huang, Yingtong Lin.

**Writing – review & editing:** Guoming Chen, Yijun Chen, Li Wei.

Li Wei orcid: 0000-0003-4682-0584.
